# Does directly administered antiretroviral therapy represent good value for money in sub-Saharan Africa? A cost-utility and value of information analysis

**DOI:** 10.1371/journal.pone.0191465

**Published:** 2018-01-23

**Authors:** Rashidah T. Uthman, Andrew J. Sutton, Louise J. Jackson, Olalekan A. Uthman

**Affiliations:** 1 Health Economics Unit, Institute of Applied Health Research, College of Medical and Dental Sciences, University of Birmingham, Edgbaston, Birmingham, United Kingdom; 2 Health Economics Unit, Leeds Institute of Health Sciences, University of Leeds, Leeds, United Kingdom; 3 Warwick-Centre for Applied Health Research and Delivery (WCAHRD), Division of Health Sciences, Warwick Medical School, The University of Warwick, Coventry, United Kingdom; The Ohio State University, UNITED STATES

## Abstract

**Background:**

Successful antiretroviral therapy (ART) relies on the optimal level of ART adherence to achieve reliable viral suppression, avert HIV drug resistance, and prevent avoidable deaths. It has been shown that there are various groups of people living with HIV at high-risk of non-adherence to ART in sub-Saharan Africa. The objective of this study was to examine the cost effectiveness and value-of-information of directly administered antiretroviral therapy (DAART) versus self-administered ART among people living with HIV, at high risk of non-adherence to ART in sub-Saharan Africa.

**Methods and findings:**

A Markov model was developed that describes the transition between HIV stages based on the CD4 count, along with direct costs, quality of life and the mortality rate associated with DAART in comparison with self-administered ART. Data used in the model were derived from the published literature. A health system perspective was employed using a life-time time horizon. Probabilistic sensitivity analysis was performed to determine the impact of parameter uncertainty. Value of information analysis was also conducted. The expected cost of self-administered ART and DAART were $5,200 and $15,500 and the expected QALYs gained were 8.52 and 9.75 respectively, giving an incremental cost effectiveness ratio of $8,400 per QALY gained. The analysis demonstrated that the annual cost DAART needs to be priced below $200 per patient to be cost-effective. The probability that DAART was cost-effective was 1% for a willingness to pay threshold of $5,096 for sub-Saharan Africa. The value of information associated with the cost of DAART and its effectiveness was substantial.

**Conclusions:**

From the perspective of the health care payer in sub-Saharan Africa, DAART cannot be regarded as cost-effective based on current information. The value of information analysis showed that further research will be worthwhile and potentially cost-effective in resolving the uncertainty about whether or not to adopt DAART.

## Introduction

The rate of infection with HIV/AIDS is very high among people living in sub-Saharan African; since the start of the epidemic, there has been a rapid spread of this virus [1]. The global population of people living with HIV in 2013 was approximately 35 million, with approximately 70% residing in sub Saharan Africa [2]. There are approximately 24.7 million HIV/AIDS infected individuals living in sub-Saharan Africa, with women making up 58% of this population [1]. Overall, 92% of the global population of HIV infected pregnant women live in this region, and 90% of children infected with HIV globally reside in sub-Saharan Africa [3]. Approximately 1.5 million newly infected HIV individuals were diagnosed in 2013 in sub Saharan Africa, with women, accounting for 25% of new HIV infections [2]. Over 1 million HIV/AIDS infected individuals die every year in Africa, and in sub-Saharan Africa; in 2013 there were approximately 1.1 million deaths due to HIV/AIDS [2].

Antiretroviral therapy (ART) is the main established standard treatment for people infected with HIV, and has been shown to substantially improve the health status of infected individuals [4–7]. Successful ART relies on the optimal level of ART adherence, to achieve a reliable viral suppression, avert HIV drug resistance and prevent avoidable deaths [8–10]. However, adherence to ART has been a significant challenge for HIV infected individuals on such treatment, and this is more severe among people who are classified as having a high risk of non-adherence to medication, including prisoners, illicit drug users, and homeless individuals [8, 10–12]. Various interventions have been adopted to improve medication adherence among people living with HIV, such as education and counselling, patient reminders, regimen simplifications and social support [5, 13] without definitive success. Directly observed therapy has been successfully applied for supporting adherence among tuberculosis patients where it has been shown to bring about health improvements, and is an approach approved by the World Health Organization (WHO) [14]. Two systematic reviews have examined the effectiveness of directly administered ART (DAART) versus self-administered ART in improving virologic suppression of people living with HIV. However, the results of the two reviews are contradictory. While Ford et al, included only RCTs and reported that “DAART seems to offer no benefit over self-administered ART”; Hart et al included both RCTs and controlled trials and reported that “DAART had a significant effect on virologic outcomes” [5, 15].

While the existing literature has focused on the effectiveness of DAART, to the best of our knowledge, there have been no attempts to assess the likely cost-effectiveness of DAART interventions for promoting adherence to antiretroviral therapy amongst those at high-risk of non-adhering from a sub-Saharan African perspective. Without objective information about the current cost-effectiveness of DAART, it is difficult to plan substantial public health interventions to improve ART adherence. Therefore, the objective of this study was to examine the cost effectiveness DAART versus self-administered ART among people living with HIV, at high risk of non-adherence to ART in sub-Saharan Africa.

## Methods

### Model structure and assumptions

**Fig 1:**
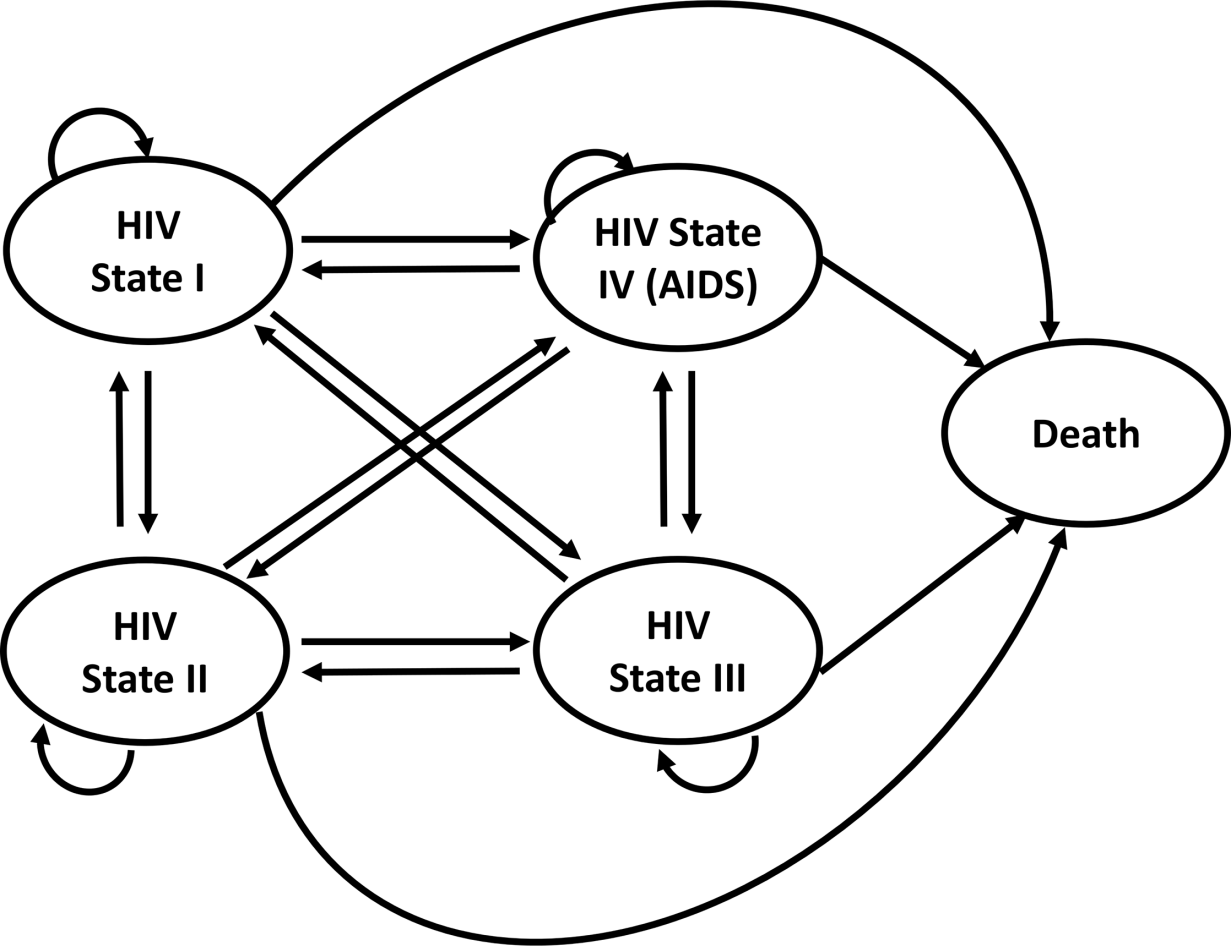
Model structure of the different model states.

A model based cost-utility analysis was undertaken to evaluate the costs and benefits associated with DAART in comparison with self-administered ART using a life time Markov model with half cycle correction [16, 17]. The study patient population was HIV-positive infected adults in sub-Saharan Africa, with a CD4 count > 500mm^3^, classified as being at high risk of non-adherence to ART. Patients were assumed to be 20 years old (the impact of this assumption was examined through sensitivity analysis) and the cycle length was one year. The perspective of government health care payers in a sub-Saharan Africa setting was taken, with only direct costs incurred by the health care provider being included in the model. The model structure consists of five HIV health states, based on the clinical categories of patient CD4 cell counts (**Fig 1**): State I, is HIV infected adults with a CD4 count of greater than 500 cells/mm^3^, without any symptoms; State II is CD4 counts of greater than 350 cells/mm^3^ but less than or equal to 500mm^3^, and is categorised as mild asymptomatic; State III is CD4 counts greater than 200 cells/mm^3^, but less than or equal to 350 cells/mm^3^, categorised as symptomatic; State IV is CD4 counts less or equal to 200 cells/mm^3^, categorised as severe symptomatic /AIDS; and State V is death. Patients can remain in the same state, or transition to any worse state or better. This approach is supported by evidence which has demonstrated the possibility that patients with AIDS can improve and move to a better HIV state, including the HIV asymptomatic state [18]. Half cycle correction was applied to costs and benefits [16, 17]. Costs and utilities were discounted at 3% [19, 20]. We used the WHO-CHOICE threshold of US$5,086 as the basis for assessments of cost-effectiveness [19, 20].

### Model input parameters

Data used in the model were derived from the published literature and are summarised in **Table 1** and described below.

**Table 1:** Model input parameters.

Parameter	Base case	Range	Reference
**EFFECTIVENESS**			
Treatment effect of DAART compared to self-administered ART (relative risk)	1.29	1.12 to 1.48	[5]
**COST**			
Annual HIV-related treatment costs (US$2014)*			
State I	176.44	383 to 639	[24, 28, 29]
State II	347.44	383 to 639	[24, 28, 29]
State III	498.44	404 to 674	[24, 28, 29]
State IV (AIDS)	1272.44	997 to 1661	[24, 28, 29]
Cost of DAART	964	723 to 1205	[30]
**UTILITY**			
State I	0.94	0.92 to 1.00	[39]
State II	0.89	0.80 to 1.00	[24]
State III	0.83	0.45 to 1.00	[24]
State IV	0.73	0.24 to 0.80	[24]
**TRANSITION PROBABILITIES**			
State I to State II	0.227	0.187 to 0.271	[21]
State I to State III	0.086	0.061 to 0.118	[21]
State I to State IV	0.062	0.040 to 0.090	[21]
State I to Death	0.022	0.010 to 0.042	[21]
State II to State I	0.195	0.163 to 0.230	[21]
State II to State III	0.202	0.170 to 0.238	[21]
State II to State IV	0.099	0.076 to 0.127	[21]
State II to Death	0.034	0.021 to 0.053	[21]
State III to State I	0.053	0.037 to 0.074	[21]
State III to State II	0.171	0.142 to 0.203	[21]
State III to State IV	0.237	0.204 to 0.273	[21]
State III to Death	0.043	0,028 to 0.063	[21]
State IV to State I	0.021	0.011 to 0.037	[21]
State IV to State II	0.095	0.072 to 0.122	[21]
State IV to State III	0.174	0.144 to 0.208	[21]
State IV to Death	0.106	0.082 to 0.134	[21]
Discount rate for costs and utilities (%)	3	0 to 5	[19, 20]

DAART: Directly administered ART

*Annual HIV-related treatment cost per clinical state is sum of cost of ART, in-patient and out-patient treatment costs, laboratory tests and the treatment of opportunitistic infections.

### Transition probabilities

All the patients were assumed to start in ‘State I’ and thus have a CD4 count greater than 500 [21]. For HIV/AIDS disease progression under the self-administered ART arm, data were used from patients that were followed-up between 2005 and 2009 from a referral hospital in Ethiopia (**Table 1**) [21]. The transition probabilities between health states in the directly administered arm were based on an estimate of the treatment effect of DAART compared with self-administered ART (relative risk) derived from a recent meta-analysis of 14 controlled trials (both randomized and non-randomized) [5]. The effect of this relative risk (RR) was calculated for each transition probability as follows [22]:

1. Increase the transition probability from one state to any better state:
  DAARTonestatetoanygoodstate=SAARTxRR
2. Reduce the transition probability from one state to any worse state:
  DAARTonestatetoanyworsestate=SAARTx1/RR.

Age-specific all-cause (not due to HIV/AIDS) mortality was applied to each state in the model (**Table 2**), and was calculated using the age-specific mortality rate from sub-Saharan Africa. This was applied in addition to the death rate from HIV/AIDS [23].

**Table 2:** ‘Natural’ age-specific mortality, South Africa 2015 projection.

Age (years)	Probability of death
10	0.0021
15	0.0020
20	0.0034
25	0.0054
30	0.0087
35	0.0143
40	0.0177
45	0.0190
50	0.0220
55	0.0263
60	0.0320
65	0.0414
70	0.0591
75	0.0830
80	0.1138

### Utilities

All the utility values for each HIV/AIDS health were derived from a survey conducted in South Africa using validated questionnaires, with HIV patients at different stages of HIV disease progression (**Table 1**) [24–27].

### Costs

The costs in this analysis included treating opportunistic infections, and all the costs of inpatient and outpatient health care utilization (**Table 1**) [24, 28–31]. The annual inpatient days and outpatient visits were calculated based on annual mean inpatient days and outpatient visits reported in the literature (**Table 1**)[32]. All the costs are converted to USA dollars, and inflated to 2014 prices [33].

### Sensitivity analyses

A series of sensitivity analyses were carried out to examine the impact on the model results of the uncertainty in the model input parameters. Structural uncertainty was assessed by assuming that no reverse progression of HIV/AIDS patients was possible (i.e. from a bad state to a better state). One way sensitivity analysis was performed, by increasing and decreasing all the input parameters by 25%, in order to examine which parameters have the greatest influence on the model results. Probabilistic sensitivity analysis (PSA) was performed with the model parameters varied simultaneously according to pre-specified distributions. Dirichlet distributions were used for all transitions with more than 2 possible outcomes. Beta distributions were used for all transitional probabilities with only two options, utilities and HIV state transition probabilities. Gamma distributions were assigned to all costs and a lognormal distribution was assigned to the effectiveness relative risk for DAART. 10,000 Monte Carlo simulations were implemented with the results shown using a cost-effectiveness acceptability curve which provides a measure of the likelihood that a decision to apply a given intervention is cost effective across a range of ‘willingness-to-pay’ thresholds, where the willingness-to-pay represents the maximum amount decision makers are willing to pay for a unit of QALY gain.

### Value of information analysis

The (individual patient) expected value of perfect information (EVPI) was calculated as the difference between the expected net health benefit (NHB) given full information and the expected NHB given current information. The NHB measure is the increase in effectiveness multiplied by the amount that the decision-maker is willing to pay per unit of increased effectiveness (i.e. the cost-effectiveness threshold), minus the increase in cost. The population EVPI was estimated by multiplying per patient EVPI by the effective population, i.e. the annual population of at high-risk of non-adhering to ART discounted over the lifetime of the treatment (assumed to be 50 years). Among people living with HIV in sub-Saharan Africa (≈24.4 million [1]) about 17.4% [34] were at high-risk of non-adhering to medication, and only 37% of people living HIV in sub-Saharan were on ART in 2013 [1]. The effective population in sub-Saharan Africa was therefore estimated at 1,600,000. The EVPI represents the maximum amount that a decision-maker should be willing to pay for further information to guide the adoption decision in the future, where additional research should be considered if the EVPI exceeds the cost of research. The expected value of partial perfect information (EVPPI) was also calculated in order to provide more focus for further research, by identifying the groups of inputs where it would be valuable to have more accurate estimates. Specifically, EVPPI estimates were generated for the following sets of parameters: effectiveness, cost, utility, and probabilities. The model used for the EVPPI was assumed to be linear for ease of computation.

## Results

### Base-case results

The base case results of the model are summarized in **Table 3.** The expected cost of self-administered ART and DAART were $5,200 and $15,500 respectively and the QALYs gained were 8.52 and 9.75 for self-administered ART and DAART respectively. The incremental cost per QALY gained for DAART versus self-administered ART was $8,400 per QALY, this suggests that DAART is not cost-effective based on the WHO-CHOICE threshold of US$5,086. The results of the simplified model assuming no reverse disease progression yielded an ICER of $9,700 per QALY.

**Table 3:** Results of cost-utility analysis.

Intervention	Cost (US$)	Incremental cost (US$)	QALYs	Incremental QALYs	ICER ((US$/QALY)
Base case^*^					
SAART	5,200	-	8.52.	-	-
DAART	15,500	10,300	9.75	1.23	8,400
**Structural uncertainty**					
SAART	6,900	-	7.83	-	-
DAART	17,600	10,700	8.94	1.11	9,700

ICER, incremental cost effectiveness ratio; PSA, Probability Sensitivity Analysis; QALY, Quality Adjusted Life Years (QALY)

Cost rounded to the nearest 100 dollars

*base case with half-cycle correction

### One-way sensitivity and threshold analyses

**Fig 2:**
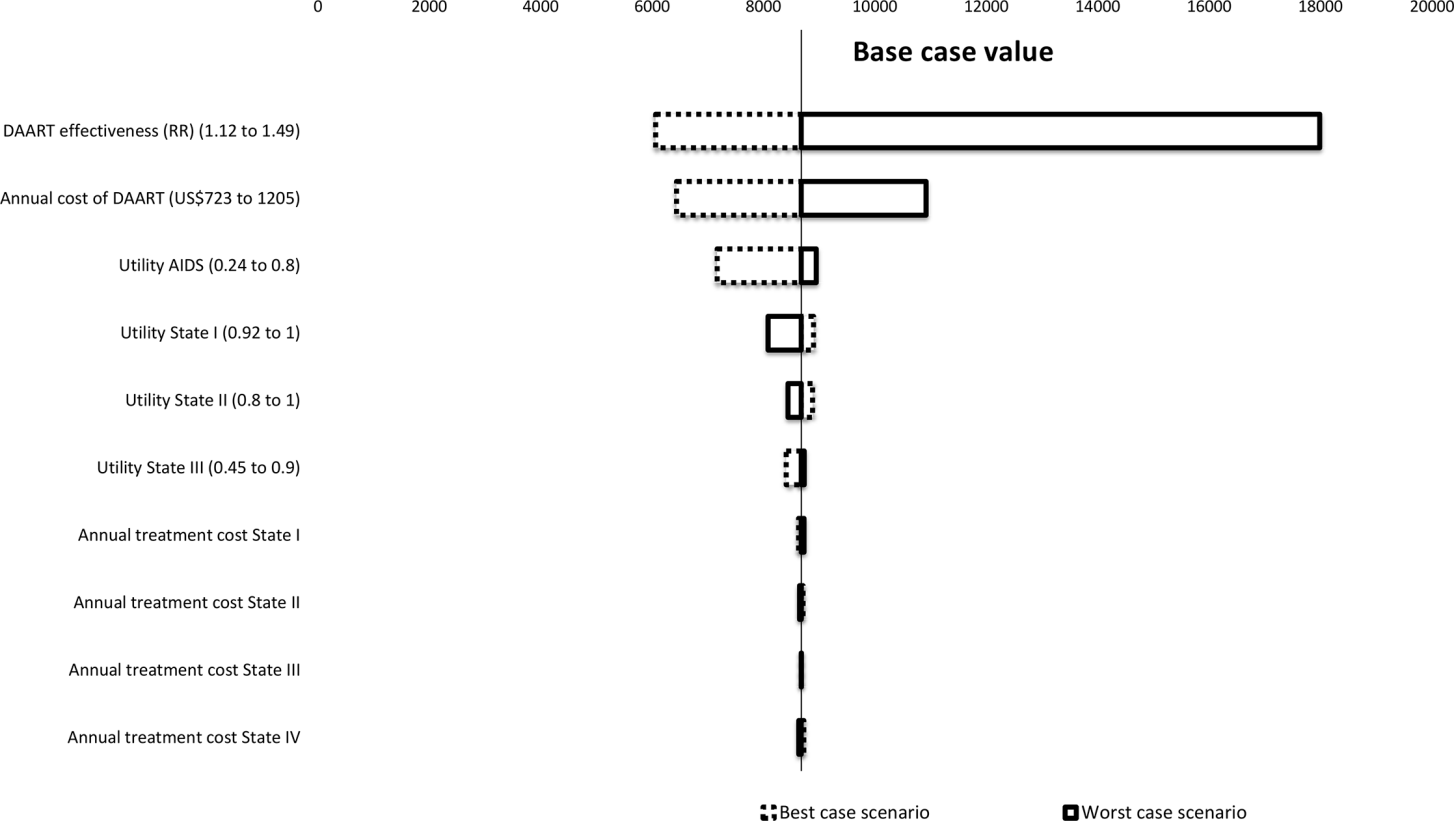
Tornado plot for the results of the one-way sensitivity analysis.

**Fig 3:**
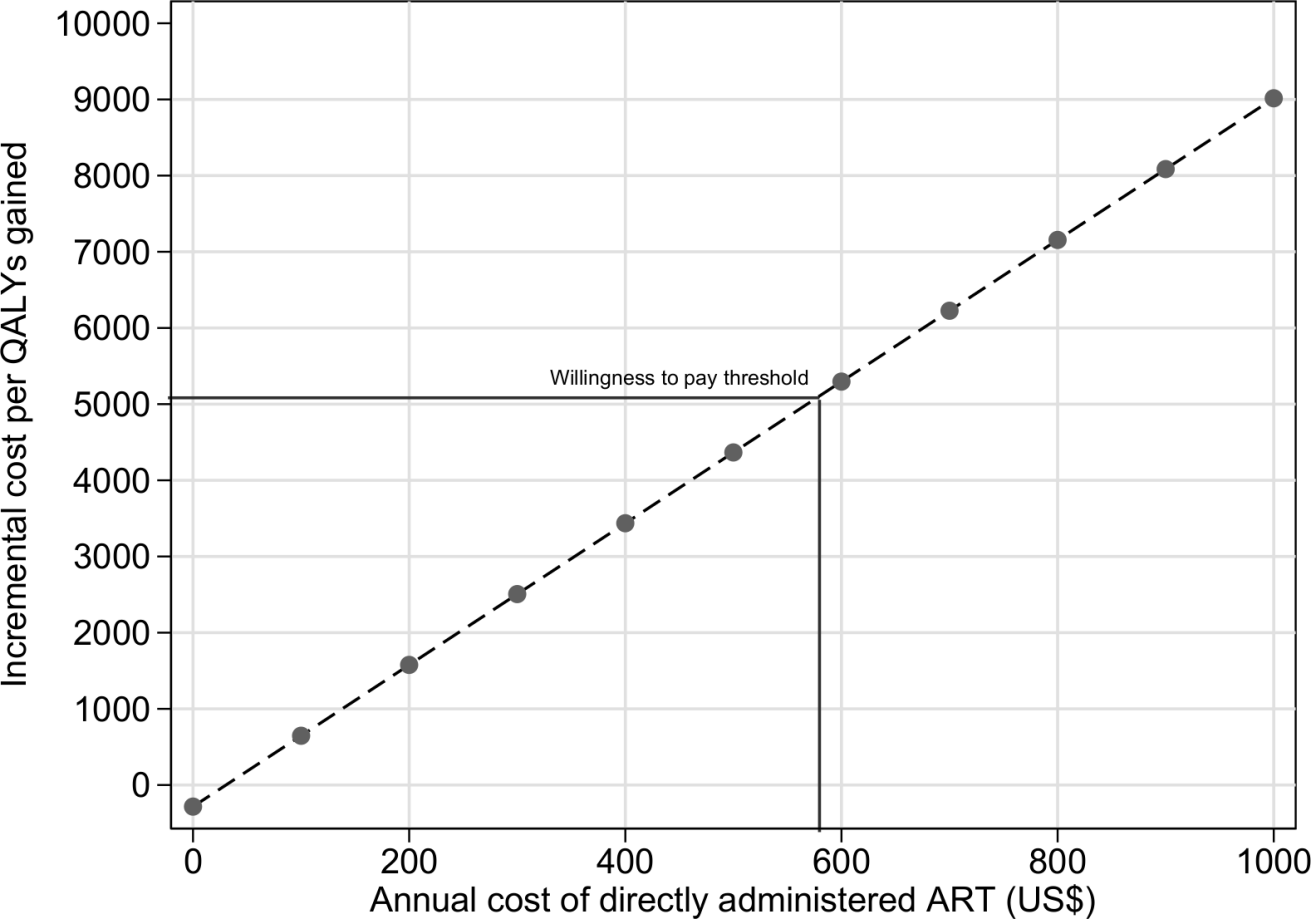
Threshold analysis for the annual cost of directly administered ART.

The results of the one-way sensitivity analysis are shown in **Fig 2**. The estimated ICER was most sensitive to variations in the cost and effectiveness of DAART. When the annual cost of DAART was varied from US$723 (best case scenario) to US$1,205 (worst case scenario) per annum, the estimated ICER ranged from US$6,440per QALY gained to US$10,921. Similarly, when the effectiveness of DAART was varied using the highest estimate from the literatre (RR = 1.49, best case scenario) and the lowest (RR = 1.12, worst case scenario), the estimated ICER was US$6,065.01 per QALY and US$17,987 per QALY respectively. The threshold analysis showed that as the annual cost of DAART increases, the cost of this treatment becomes less and less cost-effective (**Fig 3**). For DAART to be cost-effective its annual cost needs to be below US$500.

### Probabilistic sensitivity analysis

**Fig 4:**
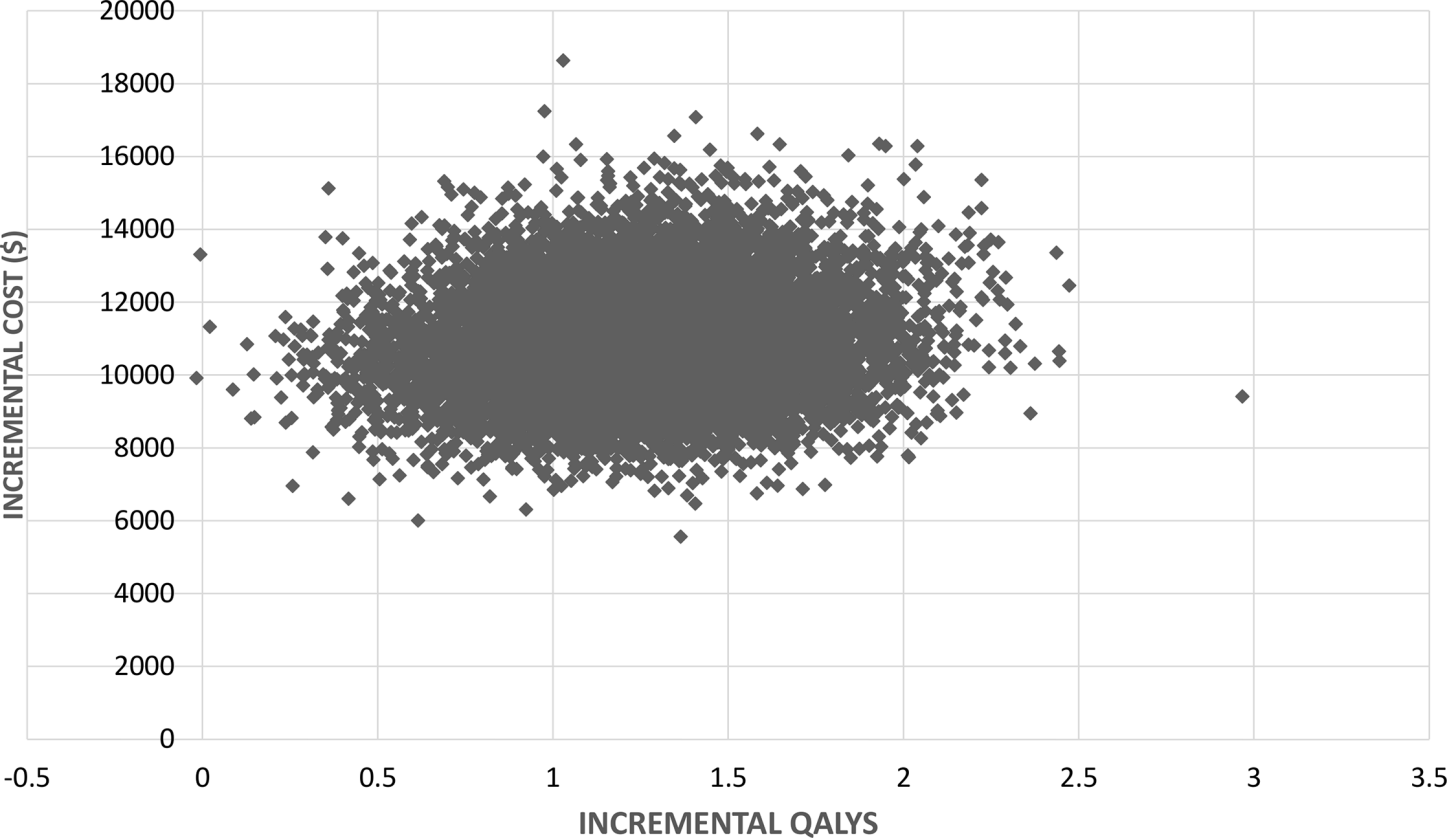
Cost-effectiveness plane for the results of the probabilistic sensitivity analysis for 10,000 simulations. Note: Reference scenario is self-administered ART.

**Fig 5:**
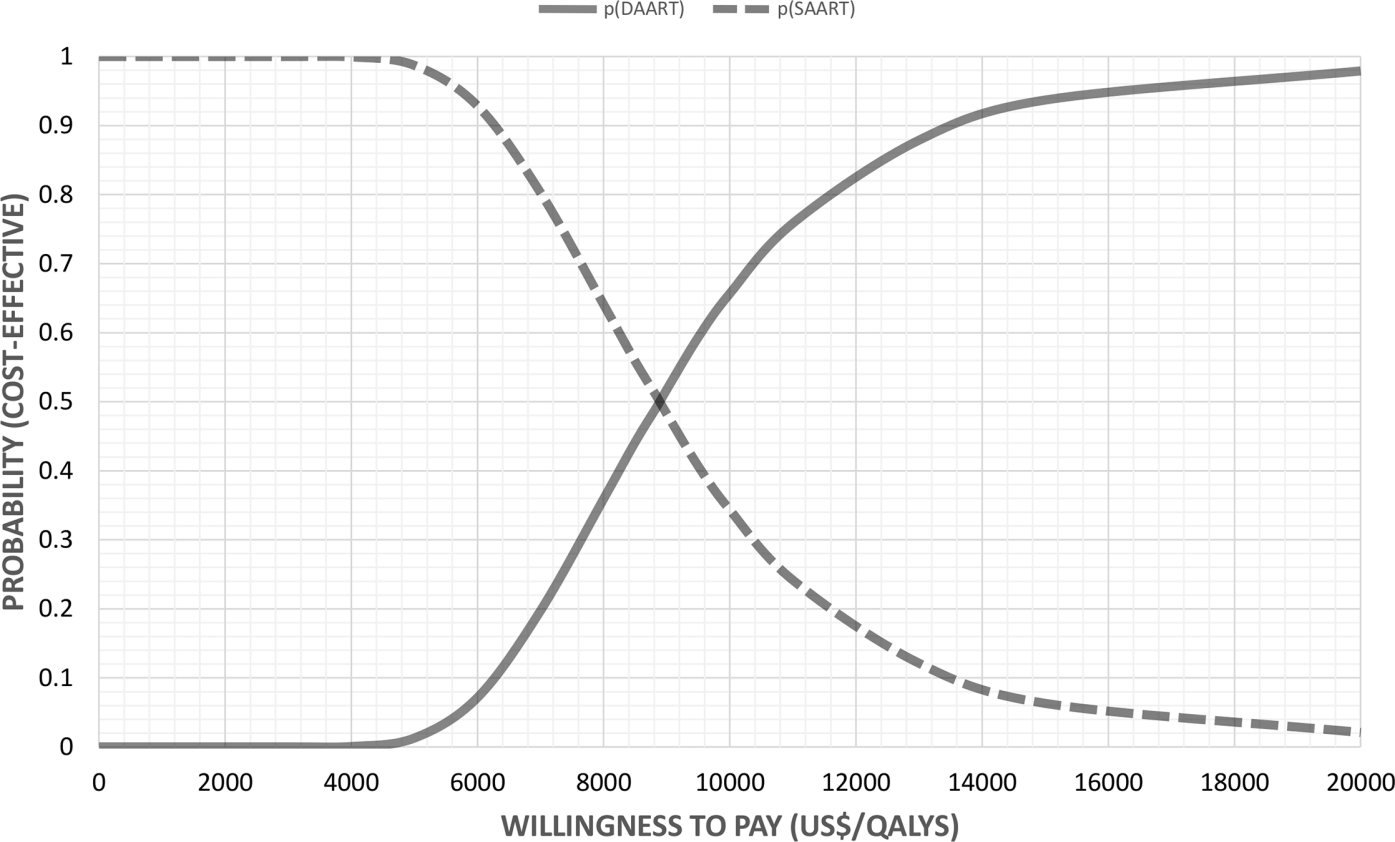
Cost effectiveness acceptability curve (CEAC) for the probabilistic sensitivity analysis for 10,000 simulations across a range of willingness to pay values for the QALY.

The output for the probabilistic sensitivity analysis for 10,000 simulations is shown in **Fig 4**. All of the model outputs were in the northeast quadrant of the cost-effectiveness plane, suggesting that DAART is more costly and more effective than self-administered ART, it is never cost saving, and never has a negative impact on patient outcomes. At a threshold of US$9,000, DAART was found to be 50% likely to be cost-effective, and if the willingness to pay for a QALY was US$18,000 then DAART is likely to be at least 95% cost-effective. The probability that DAART was cost-effective at the WHO-CHOICE threshold of US$5,086 was just 1% (**Fig 5**).

### Value of information analysis

**Fig 6:**
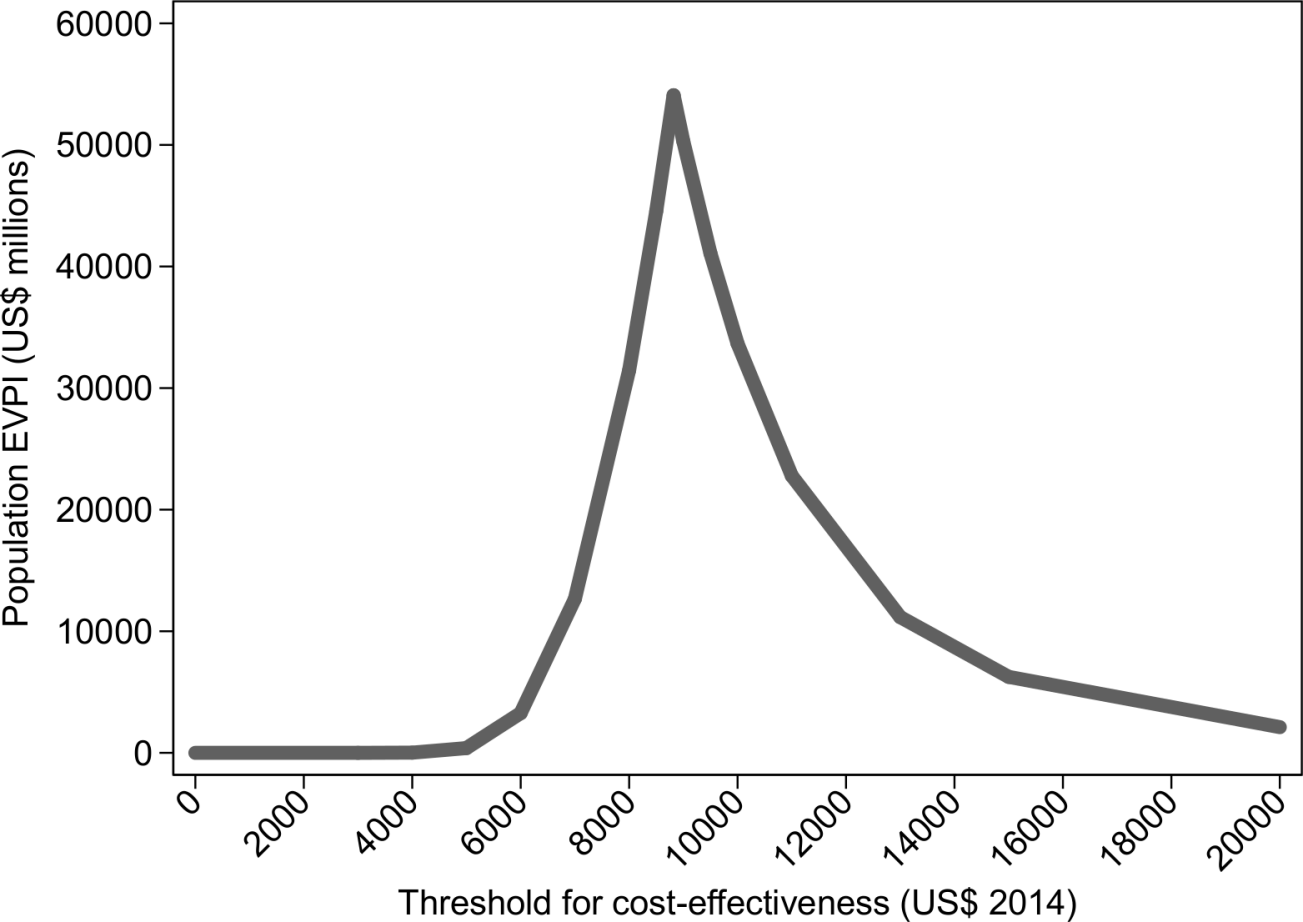
Population Expected Value of Perfect Information (EVPI).

**Fig 7:**
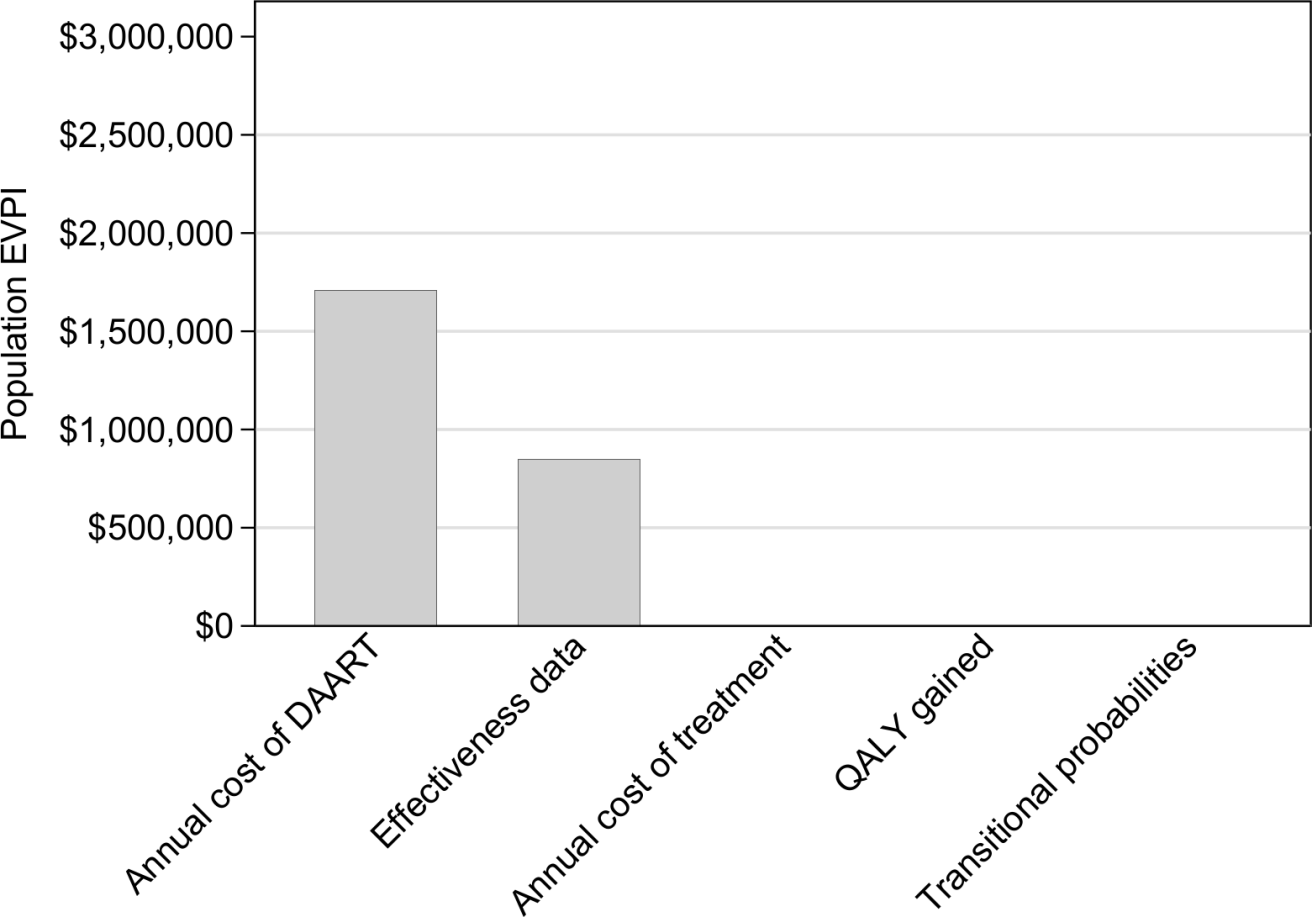
Partial Expected Value of Perfect Information (EVPI).

The population EVPI is illustrated in **Fig 6**. At cost-effectiveness threshold of $5,086, the population EVPI becomes substantial (≈US$492 million), and is likely to exceed the cost of additional investigation. This suggests that further research will be potentially cost-effective. The EVPI for each group of model parameters is illustrated in **Fig 7** for a threshold for cost-effectiveness of US$5,086. The value of information associated with research on the cost of DAART and its effectiveness is US$1,706,707 and US$853,353 respectively. The other groups of model inputs (see **Table 1**), such as transitional probabilities, QALYs gained and cost of treatment, have no value of information associated with them. This suggests that if further research is commissioned, it should focus on the relative effectiveness of DAART compared to self-administered ART and its cost.

## Discussion

### Main findings

The cost-effectiveness of DAART versus self-administered ART was analysed using a model based cost-utility analysis. The ICER of $8,700 per QALY gained indicates that when considering the WHO-CHOICE threshold of US$5,086, from the perspective of health care payer in sub-Saharan Africa, directly administered ART cannot be regarded as cost-effective. The threshold analysis conducted for a range of costs for DAART, showed that for it to be cost-effective, the annual cost of DAART needs to be priced below $500 per patient. The results from the one-way sensitivity analysis indicate that the ICER is only sensitive to the estimates of the effectiveness and annual cost of DAART. The value of information analysis showed that further primary research will be worthwhile and potentially cost-effective in resolving the uncertainty on whether or not to adopt DAART among HIV patients at high risk of non-adherence. This should focus on establishing the relative effectiveness and cost of DAART.

### Comparison with other relevant economic evaluations

To the authors’ knowledge there are no comparative studies that have examined the cost-effectiveness of DAART in a sub-Saharan Africa setting. This reflects a wider paucity of economic evidence around HIV HIV treatment in low- and middle-income countries [35]. In a closely related study that had a different patient population, McCabe and colleagues constructed a mathematical model to examine the cost-effectiveness of DAART versus self-administered ART among pregnant HIV-infected women in their third trimester in the US? [36]. Their study found that the DAART was cost-effective from the perspective of health care payer in the United States (ICER $14,233 per QALY) [36].

### Strengths and limitations

We used a model based approach [37] which had several advantages [37, 38]. It allowed the extrapolation of the outcomes reported in the literature to reflect long term consequences and thus capture important economic outcomes. The effectiveness data were extracted from a meta-analysis of multiple clinical trials instead of using data from just one clinical trial [5]. Another important strength is that the cost data were taken from recent studies. There are some limitations of this study that must be acknowledged. The main limitation pertains to the inherent uncertainty in model input parameters and the observed sensitivity of the ICER to certain key parameters. For example, some caution is required around the estimates of the cost of DAART and self-administered ART as this may vary depending on the particular health care systems in different countries. However, several sensitivity analyses were conducted to assess both the model and structural uncertainties, including one-way sensitivity analysis, threshold analysis and probabilistic sensitivity analysis. The ICER results are robust over a range of model parameters and varying most of the parameters from worst-case to best-case scenarios did not significantly impact on the results. Another important limitation is that the treatment effect estimate used for modelling DAART effectiveness as based on a meta-analysis that included trials from heterogeneous contexts (prison, methadone clinics), and only a minority of the included studies were conducted in sub-Saharan Africa. Additionally, we used evidence from the Hart meta-analysis[5] that included only randomised controlled trials which found no evidence of DAART effectiveness over the Ford meta-analysis[15] that included both the randomised controlled trials and non-randomised controlled trials.

In addition, the model adopted a simplistic representation, we did not consider the impact of DAART on transmission of HIV in the target population. The main limiting assumption of such approach includes the use of non-dynamic process at the level of the risk of each sexual encounter, which could lead to a large underestimate of benefit. Similarly, the model could underestimate cost effectiveness of DAART for prevention of HIV, because we assume no direct benefit to the female partners. We focused only on DALYs averted.

## Conclusions

The probability that DAART is cost-effective is zero if decision makers in the WHO Africa region are willing to pay less than US$5,086 per QALY gained. As such, DAART is not cost-effective based on the WHO suggested willingness to pay threshold for sub-Saharan Africa. It was also found that DAART is not cost saving, and always has a positive impact on patient outcomes compared to self-administered ART. For DAART to be cost-effective the annual cost needs to be below US$500. The value of information associated with reducing the uncertainty around the data influencing this decision is substantial, which suggests that further research will be potentially cost-effective. Further research should therefore focus on gaining further insights into the effectiveness and cost of DAART in a sub-Saharan Africa setting.
